# Association of pyridoxal 5′-phosphate (PLP) with lipid profiles: a population-based cohort study

**DOI:** 10.3389/fnut.2025.1545301

**Published:** 2025-02-26

**Authors:** Ru-yang Zhang, Yue Chen, Xin-Qi Yan, Yue Zhang, Hua Zhou, Qian Feng

**Affiliations:** ^1^Department of Neurology, Suzhou Wuzhong People’s Hospital, Suzhou, China; ^2^Department of Neurology, The Affiliated Suzhou Hospital of Nanjing Medical University, Suzhou, China

**Keywords:** PLP, lipids, LDL-C, HDL-C, vitamin B6

## Abstract

**Objectives:**

This study aims to explore the correlation between Pyridoxal 5′-Phosphate (PLP) levels and lipid profiles in adult individuals, utilizing data from the National Health and Nutrition Examination Survey (NHANES) database.

**Methods:**

The research included individuals aged 20 years and above, extracted from the NHANES database, covering the period from 2005 to 2010. The primary objective was to scrutinize the relationship between PLP and lipid profiles. This was accomplished by employing weighted, multivariable logistic regression to ascertain these associations. Furthermore, to assess the variability within different demographic segments, interaction analyses were conducted. Additionally, restricted cubic spline (RCS) methodology was implemented to delve into potential nonlinear dynamics between PLP concentrations and lipid levels.

**Results:**

A cohort of 6,459 individuals was included in this study. Our data indicated that 51.60% of the participants were under 50 years old, while 48.40% were over 50, comprising 48.83% males and 51.17% females. PLP levels demonstrated a negative correlation with low-density lipoprotein cholesterol (LDL-C) levels. After controlling for confounding variables, a one-unit increment in PLP correlates with a reduction of 17.7% in LDL-C concentrations (OR: 0.823, 95% CI: 0.823–0.824, *p* < 0.001). PLP levels exhibited a positive correlation with high-density lipoprotein cholesterol (HDL-C), which increased as PLP levels rose. After controlling for all covariates, a one-unit increase in PLP levels corresponded to a 1.952-fold enhancement in the probability of high HDL-C levels (OR: 1.952, 95% CI: 1.951–1.953, *p* < 0.001). The relationship between PLP and HDL-C levels was nonlinear. Subgroup analyses indicated that PLP levels and HDL-C concentrations are positively correlated, especially among diabetic patients and non-drinkers.

**Conclusion:**

PLP levels are inversely associated with LDL-C and positively associated with HDL-C, with stronger effects observed in diabetic patients and non-drinkers. These findings underscore the potential clinical utility of PLP supplementation as a preventive measure against cardiovascular and metabolic diseases.

## 1 Introduction

The lipid profiles consist principally of low-density lipoprotein cholesterol (LDL-C), high-density lipoprotein cholesterol (HDL-C), triglycerides (TG), total cholesterol (TC), and apolipoprotein B (Apo B), and others (1). As societies have evolved and lifestyles changed, cardiovascular diseases have emerged as leading causes of global mortality (2). Dyslipidemia is a key risk factor for atherosclerosis and cardiovascular disease (3), with LDL-C specifically implicated in atherosclerosis through its effects on endothelial dysfunction and vascular smooth muscle impairment (4). HDL-C comprises various particle subpopulations, each differing in size, shape, charge, and lipid and protein content. While HDL-C performs diverse roles and may help prevent atherosclerosis, the specific contributions of its different subpopulations remain poorly understood (5). Moreover, high triglyceride levels are increasingly associated with various health issues, including metabolic heart diseases such as ischemic heart disease (6). Elevated Apo B levels, prevalent in patients with atherosclerotic vascular alterations, are recognized as a significant risk factor for atherosclerosis and indicative of atherogenic lipoproteins (7). Therefore, elucidating the mechanisms of lipid regulation is essential for preventing and treating related diseases.

Vitamin B6 is a water-soluble vitamin that is rapidly digested and eliminated, therefore its toxicity is usually regarded minimal. However, extremely high ingestion levels can result in peripheral nerve damage (8). Research indicates that Vitamin B6 may protect against coronary heart disease (9). Oral supplementation of Vitamin B6 has been shown to mitigate lipid accumulation and dyslipidemia in Sprague–Dawley rats on a high-fat diet by reducing fatty acid and cholesterol synthesis, and enhancing fatty acid breakdown and cholesterol transport (10). PLP, the active form of Vitamin B6, participates in numerous enzymatic processes in the body, especially in amino acid metabolism (11). Plasma levels of PLP have been linked to chronic conditions such as cardiovascular disease and certain cancers, and inversely associated with various inflammation markers in both clinical and population-based studies (12). Although some research suggests that PLP may play a crucial role in fat metabolism, the specific mechanisms remain largely unexplored (13). Moreover, the relationship between PLP and lipid levels has been insufficiently investigated. Addressing this gap, this article examines the association between PLP levels and the lipid profiles of adults using the NHANES database, aiming to provide foundational evidence and theoretical support for further research.

## 2 Methods

### 2.1 Study population

This study utilized data from the Nationwide Health and Nutrition Examination Survey (NHANES), which is a regularly conducted health and nutrition survey by the Centers for Disease Control and Prevention’s National Center for Health Statistics (NCHS). The NHANES is a nationally representative, cross-sectional survey targeting non-institutionalized U.S. residents. Every 2 years, approximately 5,000 residents are randomly selected from counties across the country, with each participant assigned multiple sample weights. More details are available on the NCHS website.^1^ The NHANES protocol was approved by the National Center for Health Statistics (NCHS) Research Ethics Review Board,^2^ and written informed consent was obtained from all adult participants (14).

**Figure 1:**
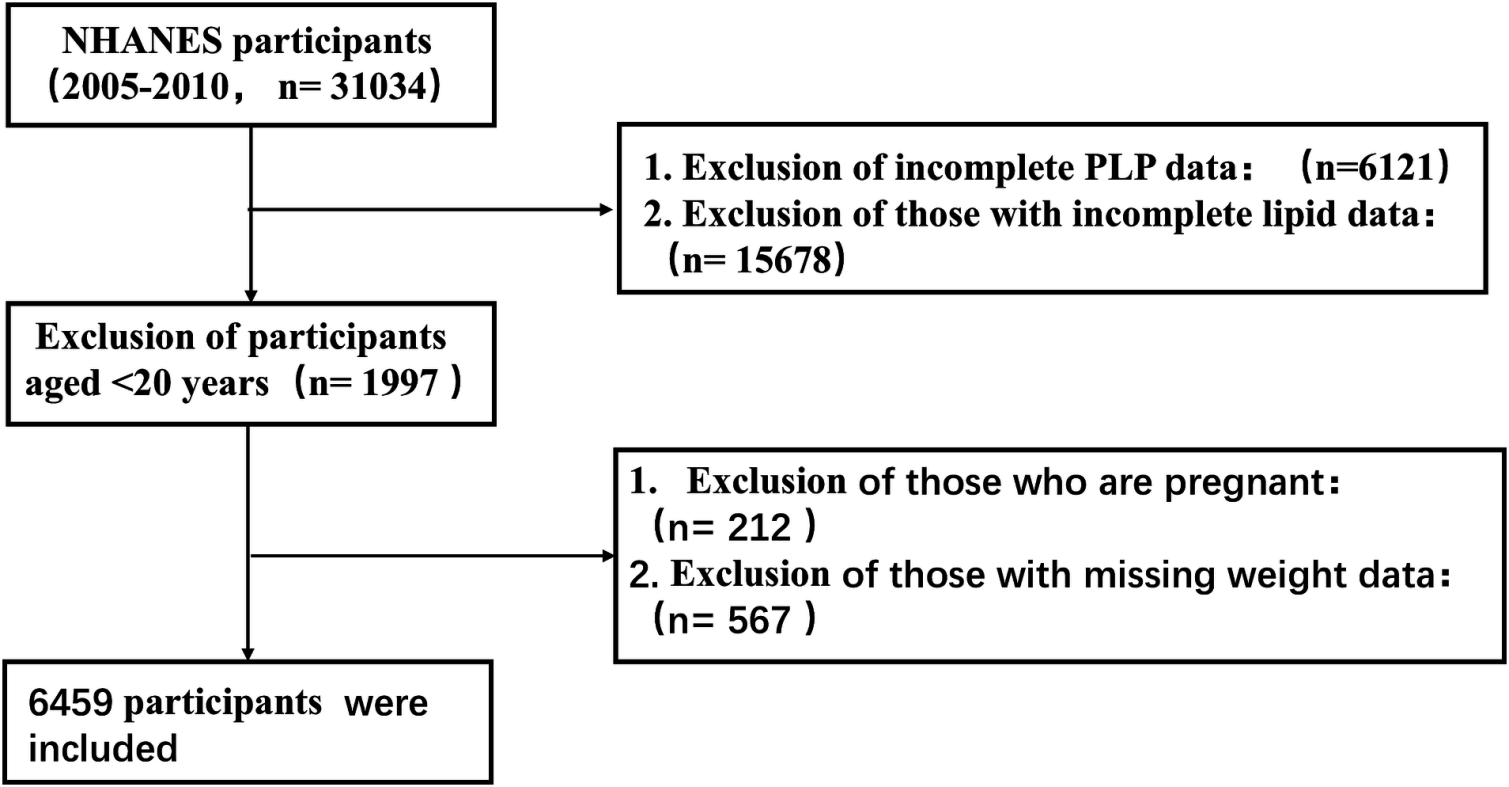
Flow chart of participant selection for the study population.

For this analysis, the dataset included U.S. adults aged 20 years and older who were part of the NHANES database from 2005 to 2010, and who had complete data on PLP and lipid profiles, excluding pregnant or lactating women. The final sample comprised 6,459 participants. Figure 1 details the participant selection process.

Participant demographics collected included age, gender, race, education level, marital status, and household poverty-to-income ratio (PIR). Clinical data encompassed body mass index (BMI), blood pressure, smoking history, alcohol consumption history, hypertension history, diabetes mellitus history, levels of PLP, LDL-C, HDL-C, TG, T, Apo B, fasting blood glucose, uric acid, glycosylated hemoglobin, fasting insulin, white blood cells, red blood cells, and hemoglobin. This information was obtained from the interview questionnaires and physical examination records in the NHANES database.

### 2.2 Detection of PLP

PLP levels were quantified using high-performance liquid chromatography (HPLC) with fluorescence detection. The mobile phase consisted of 50 mM potassium phosphate buffer (pH 3.0) and methanol, with a flow rate of 1.0 mL/min. The excitation and emission wavelengths were set to 290 nm and 395 nm, respectively.

PLP levels were measured in nmol/L.

### 2.3 Measurement of blood lipids

Blood samples were processed, stored, and transported to the University of Minnesota in Minneapolis for analysis. The biochemical analyzer at the facility was used to measure the concentrations of LDL-C, HDL-C, TG, and TC. Apolipoprotein B levels were determined through an immunochemical reaction.

### 2.4 Covariates

Beckman Coulter counting and quantification methods were used to determine the number of red blood cells, white blood cells, and hemoglobin concentration. The concentration of glycosylated hemoglobin was determined using a glycated hemoglobin analyzer. Fasting blood glucose was assessed using an enzymatic approach, fasting insulin was measured with a two-site enzyme immunoassay, and serum uric acid was quantified using a timed endpoint method. The age, gender, race, education level, and PIR were self-reported. BMI values were collected by calculating their weight and height. A smoking history was defined as having smoked at least 100 cigarettes throughout their lifetime. Drinking history was defined as more than 12 drinks per year. Hypertension is described as being informed by a doctor or other health expert that you have high blood pressure. The presence or absence of diabetes was categorized into three conditions based on participant responses: yes, no, and borderline.

### 2.5 Statistical analysis

In this study, MEC 2-year cycle weights (WTMEC2YR) were applied for statistical analysis. Missing data were addressed through multiple imputation. Continuous data were presented as mean ± standard deviation ( $\bar{x}$ ± s), while categorical variables were represented as frequency (percentage,%). When continuous numerical variables followed a normal distribution and had homogeneous variance, an independent sample t test was utilized. The Wilcoxon rank-sum test was employed for variables that did not follow a normal distribution across the two groups, while the Chi-square or Fisher’s exact test was used for categorical variables. The association between continuous numerical variables and categorical variables was examined using one-way analysis of variance. The data were divided into two groups based on the mean values of LDL-, HDL-C, TG, TC, apolipoprotein B, age, and PIR, and then transformed into categorical variables. PLP was separated into four categories based on quartile values and then converted into categorical variables. Initially, the distribution of the four groups of data was evaluated using the PLP quartile.

Weighted univariate regression was employed to analyze the relationship between each vaxbarriable and PLP. Significant variables (*p* < 0.01) were included in the further analysis models. The association between PLP and lipid profile was evaluated using weighted multivariate logistic regression. In model 1, the variables were not modified, however in model 2, they were adjusted for age, gender, race, education, marital status, PIR, history of hypertension, diabetes, smoking history, and history of alcohol intake. Model 3 adjusted the following variables: BMI, fasting insulin, fasting blood glucose, uric acid, glycosylated hemoglobin, white blood cell count, red blood cell count, and hemoglobin. In the subgroup analysis, we grouped patients according to gender, race, education, history of hypertension, history of diabetes, history of smoking, history of alcohol consumption, family PIR, and age. Heterogeneity between subgroups was further assessed by interaction analysis. Restricted cubic spline analysis (RCS) was used to assess the nonlinear association between PLP and lipids. A two-sided *p* < 0.05 was considered statistically different in all statistics. We used SPSS 26.0 software and R software (version 4.2) for data processing.

## 3 Results

### 3.1 Baseline characteristics

Table 1 illustrates that the study included a total of 6,459 participants, with 51.60% under the age of 50 and 48.40% over the age of 50. Of these participants, 48.83% were male and 51.17% were female. The participants were divided into four groups based on the quartiles of their PLP levels. With escalating PLP levels, several trends emerged: there was a rise in the percentage of males, smokers, married individuals, and those without diabetes, as well as an increase in high TC and HDL-C prevalence. Conversely, there was a decline in BMI, fasting glucose (FG), and glycated hemoglobin (HbA1c) levels.

**Table 1:** Baseline characteristic of participants.

Variables	Total (*n* = 6,459)	Q1 (*n* = 1,611)	Q2 (*n* = 1,615)	Q3 (*n* = 1,614)	Q4 (*n* = 1,619)	*p*
Age, N (%)						<0.001
<50 years	3,333 (51.60)	727 (45.13)	882 (54.61)	941 (58.30)	783 (48.36)	
≥50 years	3,126 (48.40)	884 (54.87)	733 (45.39)	673 (41.70)	836 (51.64)	
Gender, N (%)						<0.001
Male	3,154 (48.83)	608 (37.74)	762 (47.18)	915 (56.69)	869 (53.68)	
Female	3,305 (51.17)	1,003 (62.26)	853 (52.82)	699 (43.31)	750 (46.32)	
Race, N (%)						<0.001
Mexican American	1,170 (18.11)	241 (14.96)	339 (20.99)	339 (21.00)	251 (15.50)	
Other Hispanic	596 (9.23)	137 (8.50)	154 (9.54)	164 (10.16)	141 (8.71)	
Non-Hispanic White	3,155 (48.85)	760 (47.18)	758 (46.93)	761 (47.15)	876 (54.11)	
Non-Hispanic Black	1,247 (19.31)	412 (25.57)	290 (17.96)	269 (16.67)	276 (17.05)	
Other Race	291 (4.51)	61 (3.79)	74 (4.58)	81 (5.02)	75 (4.63)	
Education, N (%)						<0.001
Less Than 9th Grade2	817 (12.65)	230 (14.28)	230 (14.24)	186 (11.52)	171 (10.56)	
9-11th Grade	1,016 (15.73)	351 (21.79)	278 (17.21)	214 (13.26)	173 (10.69)	
High School Grad	1,543 (23.89)	437 (27.13)	389 (24.09)	388 (24.04)	329 (20.32)	
Some College or AA degree	1762 (27.28)	380 (23.59)	466 (28.85)	444 (27.51)	472 (29.15)	
College Graduate or above	1,321 (20.45)	213 (13.22)	252 (15.60)	382 (23.67)	474 (29.28)	
Marital Status (%)						<0.001
Married	3,455 (53.49)	756 (46.93)	851 (52.69)	913 (56.57)	935 (57.75)	
Widowed	570 (8.82)	202 (12.54)	133 (8.24)	102 (6.32)	133 (8.21)	
Divorced	671 (10.39)	215 (13.35)	171 (10.59)	135 (8.36)	150 (9.26)	
Separated	230 (3.56)	68 (4.22)	63 (3.90)	56 (3.47)	43 (2.66)	
Never married	1,035 (16.02)	246 (15.27)	259 (16.04)	272 (16.85)	258 (15.94)	
Living with partner	498 (7.71)	124 (7.70)	138 (8.54)	136 (8.43)	100 (6.18)	
Smoking, N (%)						<0.001
No	2,981 (46.15)	884 (54.87)	762 (47.18)	683 (42.32)	652 (40.27)	
Yes	3,478 (53.85)	727 (45.13)	853 (52.82)	931 (57.68)	967 (59.73)	
Drinking, N (%)						<0.001
No	5,661 (87.65)	1,370 (85.04)	1,419 (87.86)	1,439 (89.16)	1,433 (88.51)	
Yes	798 (12.35)	241 (14.96)	196 (12.14)	175 (10.84)	186 (11.49)	
Hypertension, N (%)						<0.001
No	2,333 (36.12)	723 (44.88)	586 (36.28)	489 (30.30)	535 (33.05)	
Yes	4,126 (63.88)	888 (55.12)	1,029 (63.72)	1,125 (69.70)	1,084 (66.95)	
DM, N (%)						<0.001
No	5,594 (86.61)	1,310 (81.32)	1,400 (86.69)	1,428 (88.48)	1,456 (89.93)	
Yes	748 (11.58)	268 (16.64)	191 (11.83)	156 (9.67)	133 (8.21)	
Borderline	117 (1.81)	33 (2.05)	24 (1.49)	30 (1.86)	30 (1.85)	
PIR						<0.001
<2.56	3,224 (49.91)	982 (60.96)	826 (51.15)	769 (47.65)	647 (39.96)	
≥2.56	3,235 (50.09)	629 (39.04)	789 (48.85)	845 (52.35)	972 (60.04)	
TG (mg/dL)	123.24 ± 65.66	128.15 ± 64.13	122.73 ± 64.71	124.48 ± 66.74	118.69 ± 66.33	<0.001
TC (mg/dL)	194.95 ± 39.74	193.11 ± 40.85	193.18 ± 38.62	195.5 ± 39.85	197.46 ± 39.63	<0.001
HDL-C (mg/dL)	54.62 ± 15.88	51.08 ± 14.51	53.53 ± 15.49	54.95 ± 16.06	58.06 ± 16.36	<0.001
LDL-C (mg/dL)	115.68 ± 34.96	116.39 ± 35.24	115.12 ± 34.81	115.66 ± 35.09	115.66 ± 34.75	<0.001
ApoB (mg/dL)	94.06 ± 24.39	95.22 ± 24.64	93.32 ± 24.49	93.75 ± 24.28	94.12 ± 24.18	<0.001
UA (umol/L)	5.53 ± 1.43	5.53 ± 1.52	5.49 ± 1.42	5.54 ± 1.39	5.53 ± 1.37	<0.001
Insulin (uU/mL)	12.78 ± 11.77	14.98 ± 15.74	13.27 ± 11.80	12.32 ± 9.65	10.55 ± 8.01	<0.001
HbA1c (%)	5.70 ± 1.01	5.90 ± 1.17	5.70 ± 1.01	5.64 ± 0.99	5.58 ± 0.84	<0.001
WBC (1,000 cells/uL)	6.77 ± 2.35	7.36 ± 3.38	6.82 ± 1.93	6.56 ± 1.80	6.32 ± 1.78	<0.001
RBC (million cells/uL)	4.70 ± 0.50	4.62 ± 0.51	4.71 ± 0.49	4.76 ± 0.49	4.71 ± 0.51	<0.001
HG (g/dL)	14.27 ± 1.55	13.85 ± 1.65	14.30 ± 1.53	14.50 ± 1.47	14.42 ± 1.45	<0.001
FG (mg/dL)	107.47 ± 32.36	112.51 ± 39.40	107.51 ± 32.39	106.35 ± 31.32	103.56 ± 23.84	<0.001
BMI (kg/m2)	29.00 ± 6.80	30.96 ± 8.34	29.31 ± 6.57	28.24 ± 5.84	27.49 ± 5.59	<0.001

Q1: ≤ 27.6 nmol/L, Q2: 27.6–45.3 nmol/L, Q3: 45.3–79.5 nmol/L, Q4: ≥ 79.5 nmol/L. PIR, Poverty-to-income ratio; TG, Triglycerides; TC, Total Cholesterol; HDL-C, High-density lipoprotein cholesterol; LDL-C, Low-density lipoprotein cholesterol; DM, Diabetes mellitus; ApoB, Apolipoprotein (B); UA, uric acid; HbA1c, Glycohemoglobin; WBC, White blood cell count; RBC, Red blood cell count; HG, Hemoglobin; FG, Fasting glucose; BMI, body mass Index.

### 3.2 Multifactorial logistic regression analysis

Table 2 details the results from a multivariate regression analysis examining the relationship between PLP levels and lipid profiles. PLP levels were inversely related to LDL-C levels. Specifically, Model 1 showed that an increase in PLP concentration was associated with a reduced risk of elevated LDL-C levels. After adjusting for covariates in Models 3, each one-unit increase in PLP level was associated with a 17.7% decrease in the risk of high LDL-C levels (OR: 0.823, 95% CI: 0.823–0.824, *p* < 0.001).

**Table 2:** Association of PLP with lipid profiles.

	Model 1		Model 2		Model 3	
	OR (95%CI)	*p* value	OR (95%CI)	*p* value	OR (95%CI)	*p* value
LDL-C						
Q1	Ref		Ref		Ref	
Q2	0.876 (0.875–0.876)	<0.001	0.846 (0.846–0.847)	<0.001	0.823 (0.823–0.824)	<0.001
Q3	0.865 (0.865–0.866)	<0.001	0.835 (0.834–0.835)	<0.001	0.81 (0.81–0.811)	<0.001
Q4	0.936 (0.935–0.936)	<0.001	0.894 (0.894–0.895)	<0.001	0.872 (0.871–0.872)	<0.001
HDL-C						
Q1	Ref		Ref		Ref	
Q2	1.368 (1.367–1.368)	<0.001	1.526 (1.525–1.527)	<0.001	1.413 (1.413–1.414)	<0.001
Q3	1.684 (1.683–1.684)	<0.001	2.097 (2.096–2.098)	<0.001	1.891 (1.89–1.892)	<0.001
Q4	2.122 (2.121–2.123)	<0.001	2.234 (2.233–2.236)	<0.001	1.952 (1.951–1.953)	<0.001
TG						
Q1	Ref		Ref		Ref	
Q2	0.838 (0.838–0.838)	<0.001	0.91 (0.91–0.911)	<0.001	0.984 (0.983–0.984)	<0.001
Q3	0.937 (0.937–0.938)	<0.001	1.123 (1.123–1.124)	<0.001	1.252 (1.251–1.253)	<0.001
Q4	0.75 (0.749–0.75)	<0.001	0.98 (0.98–0.981)	<0.001	1.13 (1.13–1.131)	<0.001
TC						
Q1	Ref		Ref		Ref	
Q2	0.996 (0.996–0.997)	<0.001	1.04 (1.039–1.04)	<0.001	0.984 (0.983–0.984)	<0.001
Q3	1.111 (1.11–1.111)	<0.001	1.224 (1.223–1.224)	<0.001	1.163 (1.163–1.164)	<0.001
Q4	1.288 (1.287–1.288)	<0.001	1.366 (1.366–1.367)	<0.001	1.294 (1.293–1.295)	<0.001
Apo B						
Q1	Ref		Ref		Ref	
Q2	0.904 (0.904–0.905)	<0.001	0.923 (0.922–0.923)	<0.001	0.989 (0.988–0.989)	<0.001
Q3	0.857 (0.857–0.858)	<0.001	0.897 (0.896–0.897)	<0.001	1.163 (1.163–1.164)	<0.001
Q4	0.909 (0.909–0.909)	<0.001	0.995 (0.994–0.995)	<0.001	1.294 (1.293–1.295)	<0.001

Model 1: adjusted for no variable. Model 2: adjusted for age, gender, race, education, marital status, PIR, drinking, BMI, smoking, hypertension, DM. Model 3: adjusted for age, gender, race, education, marital status, PIR, drinking, BMI, smoking, hypertension, DM, UA, Insulin, HbA1c, WBC, RBC, HG, FG. OR, Odds Ratio; CI, Confidence Interval. PIR, Poverty-to-income ratio; TG, Triglycerides; TC, Total Cholesterol; HDL-C, High-density lipoprotein cholesterol; LDL-C, Low-density lipoprotein cholesterol; DM, Diabetes mellitus; ApoB, Apolipoprotein (B); UA, uric acid; HbA1c, Glycohemoglobin; WBC, White blood cell count; RBC, Red blood cell count; HG, Hemoglobin; FG, Fasting glucose; BMI, body mass Index.

Furthermore, PLP levels were positively associated with HDL-C levels, which increased as PLP concentrations rose. In Model 3, after adjusting for all covariates, each unit increase in PLP level led to a 0.952-fold increase in the likelihood of elevated HDL-C levels (OR: 1.952, 95% CI: 1.951–1.953, *p* < 0.001).

For TG, the relationship varied across different models and quartiles: In Model 1, PLP levels were negatively associated with TG levels. In Model 2, negative correlations were observed in quartiles Q2 and Q4, but a positive correlation appeared in quartile Q3. In Model 3, a negative correlation persisted in the Q2 group, while positive correlations emerged in the Q3 and Q4 groups.

Regarding TC, correlations also differed by model and quartile: In Model 1, an inverse relationship was seen in the Q2 group, while positive associations were noted in the Q3 and Q4 groups. In Model 2, PLP levels were positively associated with TC levels. In Model 3, PLP levels showed a negative correlation in the Q2 group but positive correlations in the Q3 and Q4 groups.

Apo B levels had variable correlations with PLP across the models: Models 1 and 2 demonstrated a negative association. Models 3 showed negative correlation in the Q2 group, positive correlation in the Q3 and Q4 group.

**Figure 2:**
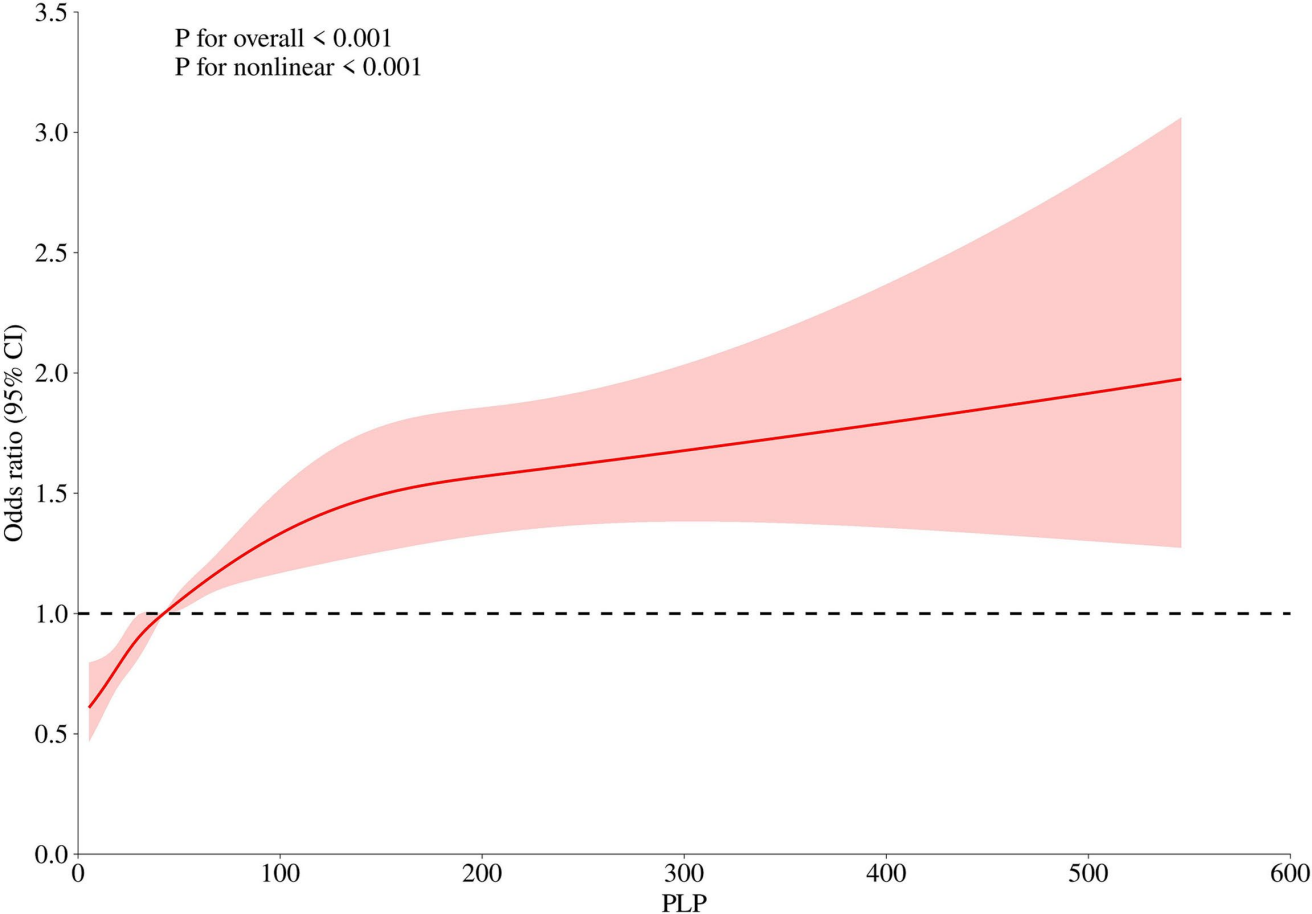
Nonlinear association between PLP Levels and HDL-C (restricted cubic spline analysis).

In the different models, PLP levels were all negatively correlated with LDL-C levels, and PLP levels were all positively correlated with HDL-C levels; as PLP levels increased, HDL-C levels increased. Therefore, we performed RCS analysis targeting the correlation between PLP level and HDL-C level. Figure 2 illustrate this nonlinear correlation.

### 3.3 Subgroup analysis: the correlation between PLP level and HDL-C level

**Figure 3:**
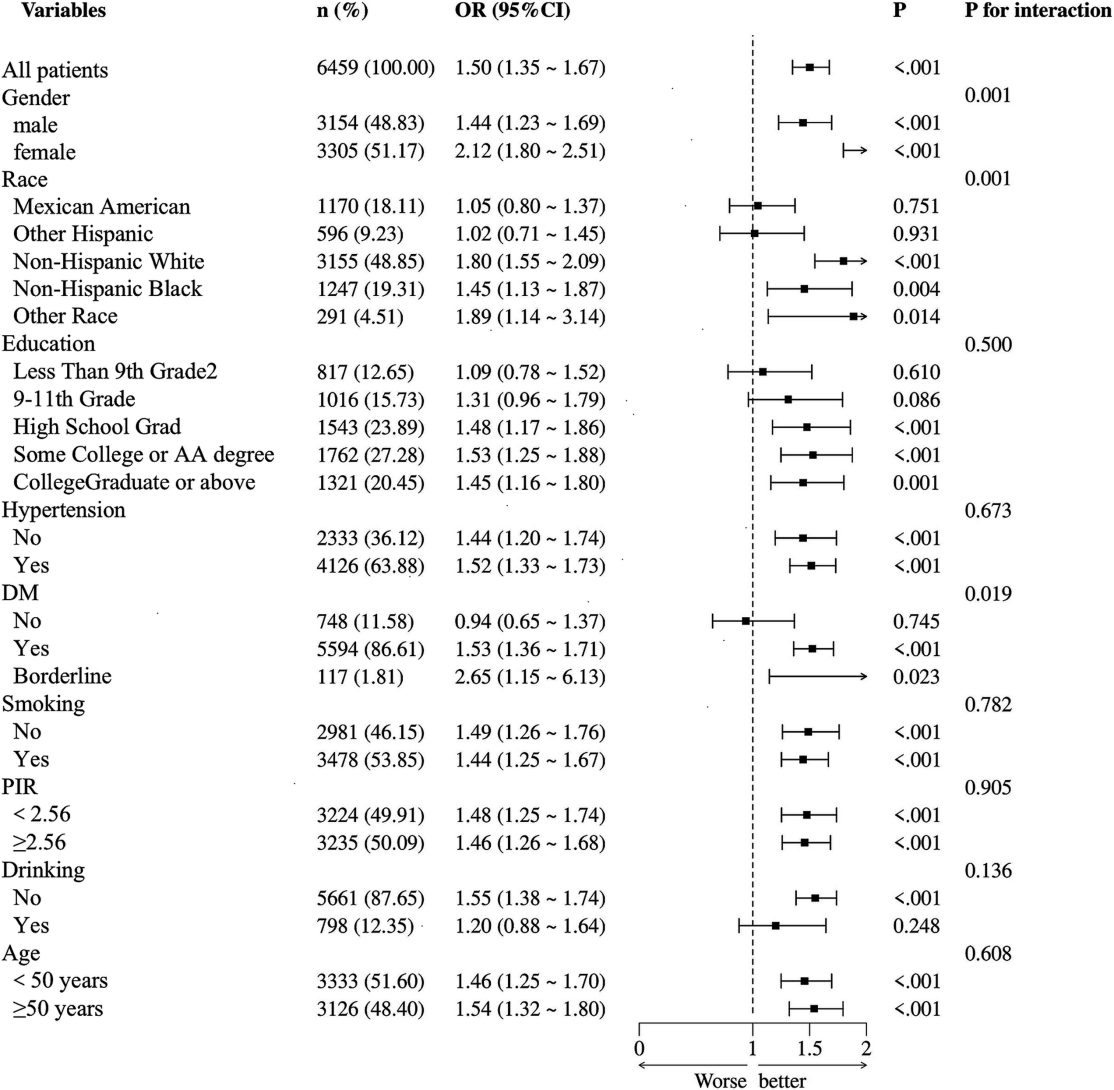
Subgroup analysis of the association between PLP quartiles and HDL-C levels. X-axis: PLP concentration (nmol/L), Y-axis: odds ratio for high HDL-C.

Subgroup analyses were conducted to verify the stability of the results. PLP was positively correlated with HDL-C, especially in non-drinkers and diabetics. As illustrated in Figure 3, PLP levels interacted with gender, race, and diabetes status, but no interactions were found with age, education level, hypertension, smoking, alcohol consumption, or PIR.

## 4 Discussion

This study utilized the NHANES database to examine the relationship between PLP levels and lipid profiles in U.S. adults. In three models, PLP levels were found to have a negative correlation with LDL-C and a positive correlation with HDL-C. HDL-C levels increased with higher PLP concentrations, particularly among individuals with diabetes and non-drinkers, and a nonlinear relationship between PLP and HDL-C was observed. After adjusting for all covariates, each one-unit increase in PLP was associated with a 12.8% reduction in the likelihood of elevated LDL-C (OR: 0.872, 95% CI: 0.871–0.872, *p* < 0.001) and a 1.952-fold increase in the likelihood of elevated HDL-C (OR: 1.952, 95% CI: 1.951–1.953, *p* < 0.001). Additionally, PLP levels were associated with total cholesterol, triglycerides, and Apo B, although these relationships were not consistent across models. These findings suggest that PLP may play a significant role in regulating lipid metabolism.

Vitamin B6 exists in various forms within our diet, yet only PLP functions as an enzyme cofactor. Non-phosphorylated forms of vitamin B6 are absorbed in the intestine and subsequently converted into the active PLP form by specialized enzymes. PLP-dependent processes include amino acid and neurotransmitter metabolism, folate and one-carbon metabolism, protein and polyamine synthesis, carbohydrate and lipid metabolism, mitochondrial function, and erythropoiesis (15).

This study is the first to specifically investigate the relationship between PLP levels and lipid profiles. Previous studies have focused on the impact of vitamin B6 supplementation on blood lipid levels. In one study, 12 dialysis patients receiving vitamin B6 and folic acid supplementation over 68 months exhibited significantly lower LDL-C levels, though TC, TG, and HDL-C levels remained largely unchanged (16). Vitamin B6 has also been shown to provide protective effects against coronary heart disease, particularly among women, individuals with obesity, and smokers (9). Additionally, vitamin B6 has been found to improve non-alcoholic fatty liver disease in mice through the PPAR and TLR4/NF-κB signaling pathways (17).

In our study, PLP levels were positively associated with HDL-C levels, with a stronger correlation observed in diabetic patients. This aligns with previous research showing that PLP deficiency impairs insulin secretion in rats, whereas PLP supplementation helps prevent diabetic complications and improves outcomes in gestational diabetes (18). Vitamin B6 has also been found to reduce insulin resistance, which is often induced by lipogenesis and fat distribution in obese individuals. Diabetic patients tend to have larger adipocytes compared to non-diabetics; insulin sensitivity is inversely related to adipocyte size (19, 20), and markers of insulin resistance are significantly associated with lower HDL-C levels (21).

Our study further indicated an inverse relationship between PLP levels and LDL-C levels. Although previous studies have not provided direct evidence of a definitive association, some suggest that low vitamin B6 intake may be linked to an increased risk of cardiovascular disease. One study reported that individuals with plasma PLP levels below 30 nmol/L had a significantly higher risk of coronary heart disease than those with levels above 30 nmol/L. Additionally, the combination of low PLP and abnormal lipid levels was associated with an even greater risk of coronary heart disease (22). Recent research has found that serum PLP levels are negatively correlated with all-cause mortality, cardiovascular mortality, and CVD risk in US adults, with a dose–response relationship (23). Other studies have produced similar findings (24). Some researchers, however, noted that the association between low plasma PLP levels and poor cardiovascular outcomes may differ by gender, with a stronger and more independent link observed in women (25). However, a study has found that there is no relationship between PLP and the risk of myocardial infarction (26). Considering these research findings, the relationship between PLP and cardiovascular diseases is complex and multifaceted, warranting further studies to elucidate their specific mechanisms and impacts.

The factors influencing PLP levels are not fully established. Aging and smoking are known to reduce plasma PLP concentrations, while moderate alcohol consumption may amplify these effects. Some studies suggest that the influence of moderate alcohol intake on plasma PLP levels may be partly due to the vitamin B6 content in beer (27). Other research has indicated no significant decrease in PLP levels between the ages of 60 and 90; however, age-related shifts in body composition, such as an increased fat-to-lean mass ratio, may negatively impact vitamin B6 status (28).Analysis of data from the National Diet and Nutrition Survey Rolling Program found that dietary B vitamin intake and plasma PLP levels decline with age and lifestyle factors, particularly among older adults who smoke and take multiple medications (polypharmacy) (29). Additionally, some studies have associated abdominal obesity with lower vitamin B6 levels (30).

The direct correlation between TC, TG, ApoB, and PLP is exceedingly limited. However, there are a few literature reports that have explored the association between vitamin B6 and these lipid indicators. In our research findings, TC, TG, and ApoB exhibit inconsistent correlations with PLP. Previous studies have indicated that supplementation with vitamin B6 does not significantly affect serum cholesterol levels (31). Nonetheless, animal studies have demonstrated that oral administration of vitamin B6 can inhibit the synthesis of fatty acids and cholesterol, promote the breakdown of fatty acids, and enhance cholesterol transport (10). Furthermore, an experiment involving Japanese quail showed that injection of vitamins C, B6, and B12 into fertilized eggs resulted in hatched quails with significantly reduced plasma total lipids and cholesterol levels (32). Additionally, a randomized controlled trial revealed that supplementation with a complex containing multiple B vitamins led to significant reductions in fasting glucose, triglycerides, and total cholesterol levels (33). These findings suggest that vitamin B6 may hold potential for regulating blood lipid levels.

Given that humans cannot synthesize vitamin B6 endogenously (34), dietary intake is critical. To optimize PLP levels, individuals should prioritize foods rich in vitamin B6, such as bonito, tuna, chicken liver, fish, and potatoes (35). For high-risk populations (diabetics or those with dyslipidemia), supplementation under medical supervision may be warranted. Additionally, smoking cessation is advised, as this factor exacerbates PLP depletion (29).

This cross-sectional study, based on a large-scale, representative survey, thoroughly controlled for confounding factors and was the first to examine the association between PLP and lipid profiles. However, several limitations should be noted: (i) Data on factors influencing lipid levels, such as lipid-lowering medication use and body weight management, were incomplete and therefore could not be included as variables. (ii) Due to the cross-sectional design and lack of follow-up data, establishing causality is challenging.

## 5 Conclusion

In summary, blood lipid levels have long been a primary concern for clinicians due to the distinct health impacts of different lipid components. Our study found that PLP levels were inversely associated with LDL-C levels and positively associated with HDL-C levels; specifically, an increase in PLP levels corresponded with higher HDL-C levels. Additionally, a nonlinear relationship between PLP and HDL-C levels was observed. While vitamin B6 or PLP supplementation appears to benefit blood lipid profiles and may aid in clinical diagnosis and treatment, further research is needed to elucidate the precise mechanisms involved.

## Data Availability

The raw data supporting the conclusions of this article will be made available by the authors, without undue reservation.

## Footnotes

^1^ https://www.cdc.gov/nchs/nhanes/index.htm

^2^ http://www.cdc.gov/nchs/nhanes/irba98.htm
